# Current and Future Implications of COVID-19 among Youth Wheelchair Users: 24-Hour Activity Behavior

**DOI:** 10.3390/children8080690

**Published:** 2021-08-11

**Authors:** Ryan T. Conners, Lauren C. Bates, Patricia Pagan Lassalle, Gabriel Zieff, Paul N. Whitehead, Sandra Stevens, Lauren Killen, Robert Cochrum, Kathryn L. Rodebaugh, Mark Faghy, Lee Stoner

**Affiliations:** 1 Department of Kinesiology, The University of Alabama in Huntsville, Huntsville, AL 35899, USA; Paul.Whitehead@uah.edu (P.N.W.); klr0039@uah.edu (K.L.R.); 2 Department of Exercise and Sport Science, University of North Carolina at Chapel Hill, Chapel Hill, NC 27599, USA; lbates15@live.unc.edu (L.C.B.); ppagan@unc.edu (P.P.L.); gzieff@live.unc.edu (G.Z.); stonerl@email.unc.edu (L.S.); 3 Department of Health and Human Performance, Middle Tennessee State University, Murfreesboro, TN 37132, USA; Sandra.Stevens@mtsu.edu; 4 Department of Kinesiology, University of North Alabama, Florence, AL 35632, USA; lkillen1@una.edu; 5 Department of Human Performance and Sport Sciences, Tennessee State University, Nashville, TN 37209, USA; rcochrum@tnstate.edu; 6 Human Sciences Research Centre, University of Derby, Derby DE22 1GB, UK; M.Faghy@derby.ac.uk

**Keywords:** youth wheelchair users, physical activity, sedentary behavior, COVID-19

## Abstract

Preventative measures taken worldwide to decrease the transmission of COVID-19 have had a tremendous impact on youth. Following social restrictions, youth with and without physical disabilities are engaging in less physical activity, more increased sedentary behavior, and poor sleep habits. Specifically, youth wheelchair users (YWU) are likely disproportionately affected by COVID- 19 and have a higher risk of contraction due to underlying comorbidities. While we cannot control all of the negative long-term implications of COVID-19 for YWU, participation in positive 24-h activity behaviors can decrease chronic disease risk and the likelihood of long-term complications resulting from infection. This commentary is to extend the discourse on the importance of 24-h activity behaviors by focusing on YWU. Specifically, we discuss the importance of chronic disease prevention, provide a brief overview of 24-h activity behaviors, and outline some of the lessons that can be learned from the COVID-19 pandemic.

## 1. Introduction

The severe acute respiratory syndrome coronavirus 2 (SARS-CoV-2) pandemic has brought about unprecedented challenges worldwide. In an attempt to deter the spread of the virus and disease associated with the virus (COVID-19), many countries implemented social and physical distancing restrictions that led to the closure of work places, schools and, recreational facilities [1]. The impact of COVID-19 restrictions on physical activity (PA) levels in adults has been mixed due to research studies reporting decreased exercise engagement [2,3,4], the maintenance of PA levels [5], or even increased exercise practice [5,6]. However, among children, these closures have had primarily a negative impact on 24-h activity behaviors (24-AB) [1], including decreased PA, increased sedentary behavior (SB), and poor sleep habits in the sedentary profiles in children [7,8,9]. These behaviors are independently and collectively associated with poor physical and mental health outcomes [10].

The purpose of this commentary is to extend the discourse on the importance of 24-AB by focusing on youth wheelchair users (YWU), where YWU can be defined as youth aged 5–17 years who are disabled as a result of musculoskeletal, neurological, cognitive, or other types of dysfunction and use a wheelchair as their main source of mobility [11]. Specifically, we discuss the importance of chronic disease prevention, provide a brief overview of 24-AB, and outline some of the lessons that can be learned from the COVID-19 pandemic. We have focused on YWU due to the high likelihood of their 24-AB being impacted by the COVID-19 social restrictions, the high risk of developing severe illness and complications following a COVID-19 infection due to underlying health conditions and co-morbidities [12,13], and the potential for improved mental and physical health outcomes with decreased SB, increased PA and/or improved sleep habits.

## 2. Impact of COVID-19 Restrictions on Physical and Mental Health

Those with pre-existing chronic diseases, including cardiometabolic diseases such as obesity, type II diabetes, and hypertension, are at heightened risk for severe complications and death following COVID-19 infection [14]. The YWU population is largely characterized by pre-existing conditions [15], not least because some type of pre-existing condition may have pre-empted and ultimately necessitated wheelchair use. These pre-existing conditions place YWU at greater risk for COVID-19-related complications [16,17]. In addition, wheelchair use creates a situation in which the child is more susceptible to negative 24-AB, all of which are linked to chronic diseases that can further exacerbate susceptibility to COVID-19-related complications [18,19,20]. As an example, consider a YWU who has a spinal cord injury whose risk for COVID-19-related complications may be increased both as a result of autonomic dysfunction associated with the spinal cord lesion, as well as the negative impacts on cardiometabolic health related to physical inactivity and SB.

The increased risk of poor health outcomes among YWU, either directly or indirectly related to COVID-19, highlights the need for focused and effective preventive health measures in this population [18]. Physicians, allied-health practitioners, mental health professionals, as well as parents and teachers should be aware of the increased susceptibility to COVID-19 related complications faced by YWU [9], and work collaboratively and creatively to ameliorate health risks through the promotion of positive lifestyle behaviors [21]. Fortunately, 24-AB are modifiable, and can and should be targeted in YWU as a means to maintain health and reduce risk of complications for COVID-19 and/or future variants/pandemics [22].

## 3. 24-h Movement Behaviors

With respect to 24-AB the most established guidelines are available for PA, followed by sleep. However, there is sufficient evidence to strongly associate each 24-AB, with chronic disease outcomes. For example, meeting PA guidelines is extremely important to improve physical and mental health as well as preventing many chronic diseases such as hypertension or diabetes. For youth, at least 60 min of moderate to high intensity physical activity is recommended per day [23,24]. Sufficient sleep duration and quality are also critical in supporting mental health, immune function, and attention span [25,26]. Therefore, 9–11 h of uninterrupted sleep is recommended for youth per night [23]. Lastly, SB is an independent risk factor for cardiometabolic diseases in adults [27] and likely youth [10]. Sedentary behavior has been defined as any waking behavior in a seated or reclining posture (<1.5 METS) [28]. However, due to the lack of available evidence regarding SB and health outcomes in wheelchair users (or individuals with physical disabilities), the most recent World Health Organization (WHO) Guidelines concluded that there is no reason to believe that recommendations to reduce SB would be any different for wheelchair users [29]. The WHO recommends reducing SB, and others recommend <2 h of screen time per day specifically for youth [23,30,31].

Activity behaviors including PA, sleep, and SB interact with one another across a 24-h day. Therefore, time spent engaging in one activity behavior should not be considered independently from the other behaviors. Time spent engaging in one activity behavior influences the physiological processes involved in the other behaviors. For example, increasing PA (e.g., using an arm ergometer) may lead to a reduction in SB or reducing TV time may result in a child going to bed earlier or improving their sleep quality [32,33]. It is extremely important for parents or guardians to establish a routine that promotes positive 24-AB in order to achieve the recommended guidelines for increasing PA, reducing SB, and promoting good sleep duration and quality.

## 4. Challenges Moving Forward and Lessons Learned

**Figure 1:**
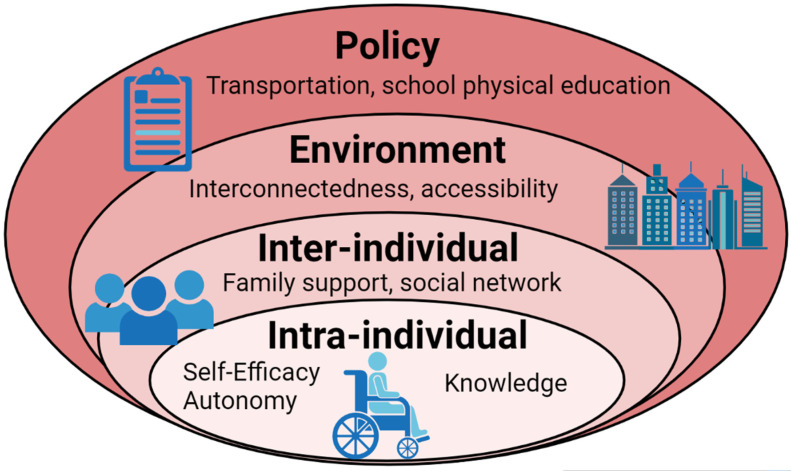
Influential factors on physical activity in youth wheelchair users: A socioecological approach.

Infection with the COVID-19 virus and the imposed social restrictions will likely have lasting health impacts on YWU [14]. At present many of the long-term health impacts cannot be predicted. Additionally, it is unclear what types of specialized healthcare these youth will require (e.g., respiratory and cardiovascular), or whether there are enough properly trained medical experts to provide the necessary acute and chronic specialized medical care. While we cannot control all of the negative long-term implications of COVID-19, there is reason to believe that positive 24-AB can be beneficial to health outcomes [33]. Additionally, we are now in a position to reflect on events surrounding the COVID-19 pandemic. Specifically, in the remainder of this section we will use a socioecological model (SEM) to provide perspective on which lessons we can learn from and make use of moving forward (see Figure 1). The SEM posits that the ability to motivate or educate an individual to change their behavior is likely to be restricted if their socio-cultural and physical environments do not enable and support the behavior [33]. Specifically, the SEM allows us to contextualize the multiple levels of influence on behavior, including intra-individual, inter-individual, physical-environment, and policy levels. The policy level is beyond the scope of this short commentary; the remainder of this section will focus on the Intra-Individual, Inter-Individual, Physical-Environment Levels.

The Intra-Individual Level includes factors such as self-efficacy and activity enjoyment. The COVID-19 pandemic has led many of us to become more self-sufficient, and to realize that we can do more with less—including engaging in PA and breaking-up SB within our homes [34]. This heightened self-reliance can be channeled to raise self-efficacy towards positive 24-AB [28]. Simple techniques include the use of goal setting, self-monitoring, and self-management [35]. This could include PA tracking via smartphone apps, setting and monitoring fixed bedtime and waketimes, and getting timed reminders to break-up sedentary behaviors [30]. Simple yet enjoyable activities that can be engaged in within the home include breaking-up sedentary behavior with light PA (e.g., playing with a pet), or participating in modified yoga available via the internet. Of relevance to the Inter-Individual Level of the SEM, YWU can participate in activities while engaging with others. To combat isolation, during the COVID-19 pandemic many people have learned to interact using various virtual platforms. The use of such platforms can continue post-COVID to, for example, challenge family or friends to SB interruption challenges or to participate in PA classes [36]. For example, individuals within support groups could challenge one another to engage musculature for at least one minute every hour or by reminding one another to break-up a sedentary bout with resistance band exercises. Additionally, positive 24-AB habits could be a family affair, including encouraging parents to restrict night-time access to screened devices (and harmful blue light), and replace the screen time with story time. Lastly, the Physical Environment-Level can be used to contextualize barriers to engaging in positive 24-AB. While COVID-19-related social restrictions have been viewed negatively with respect to our health and well-being, many individuals have adapted their home environments to improve their quality of life [37]. While physical therapy, gyms, leisure centers, and other facilities are beginning to operate on a normal schedule, the adaptations made during the pandemic need not be reversed. As opposed to physical infrastructure around the home, including paths, greenways, and public transportation, barriers within the home are relatively easy to reduce [38]. Modifications could include installing grab-bars to provide opportunities to break-up SB, or more simply placing resistance bands/other equipment around the home to make it easier to replace SB with PA. Additionally, to improve sleep-wake cycles the home environment could be modified to ensure children are positioned throughout the day to enhance exposure to sunlight, and timers can be set ensure children go outside at regular intervals.

## 5. Conclusions

Challenges faced by YWU include the greater risk of developing severe illness and complications following a COVID-19 infection, and the inability to fully predict the long-term health impacts of COVID-19 to the pandemic. However, we can take a moment to reflect and take away some important lessons gleaned during the pandemic era. For example, among the general able-bodied population we know that positive 24-AB improve chronic disease outcomes, and in doing so decreases the risk of COVID-19 infection complications. We have no reason to believe the same is not true for YWU. Using the SEM to provide context, we can take something positive away from this blight on our history, by reflecting on the adaptions we made to improve our quality of life during the pandemic to model positive and long-term 24-AB.

## Data Availability

Not applicable.
